# Nutritional knowledge, attitudes, and practices among residents in the Northeast areas of China during the COVID-19 epidemic

**DOI:** 10.3389/fpubh.2024.1296869

**Published:** 2024-01-30

**Authors:** Liyan Hou, Xueyan Xia, Ying Du, Yu Zhang, Shuangshuang Li, Wen Liu, Jie Zhao, Ke Wang, Lei Zhang, Qingshan Wang

**Affiliations:** ^1^Dalian Medical University Library, Dalian, China; ^2^National-Local Joint Engineering Research Center for Drug-Research and Development (R&D) of Neurodegenerative Diseases, Dalian Medical University, Dalian, China; ^3^Dalian Municipal Center for Disease Control and Prevention, Dalian, China; ^4^Dalian Xinyulong Marine Organisms Seed Industry Technology Co., Ltd, Dalian, China; ^5^Department of Clinical Nutrition, Second Affiliated Hospital of Dalian Medical University, Dalian, China; ^6^School of Public Health, Dalian Medical University, Dalian, China

**Keywords:** COVID-19 pandemic, SARS-CoV-2, nutrition, knowledge, attitudes, and practices, dietary behavior

## Abstract

**Background:**

The coronavirus disease 2019 (COVID-19) due to SARS-CoV-2 infection continues to affect the daily life of communities worldwide. Nutrition is a vital determinant of overall health. Given the lack of specific drugs for COVID-19 and incomplete vaccination coverage, optimizing nutrition appears to be one of the most cost-effective means of enhancing immunity. Therefore, this study was designed to evaluate nutrition-related knowledge, attitudes, and practices (KAP) to offer insights into the personal determinants of dietary behavior during COVID-19 pandemic in four major cities within the Northeast region.

**Methods:**

This cross-sectional study was conducted between January and December 2022 using a self-administered questionnaire. The data were entered in EpiData V-3.02 and analyzed using SPSS version 26. Binary logistic regression analysis was also employed to examine the association between dependent and independent variables.

**Results:**

A total of 4,092 respondents were included in the study. Most of the respondents demonstrated had inadequate nutrition knowledge, 26% of them provided ≥60% of correct answers. About one-third of the respondents were knowledgeable about the daily levels of oil, salt, milk, water, vegetables and fruits for adults. Furthermore, our results showed that 60.6% of participants held positive attitudes toward healthy eating. Additionally, only 54.6% of the participants have heathy dietary practices during COVID-19 pandemic. Binary logistic regression analysis showed that the following characteristics were associated with displaying unhealthy dietary behaviors: being men, having a lower education level, having a family income of 10,000–19,999 and more than 20,000, being resided in Harbin, Shenyang, and Changchun. Importantly, the strongest associations were observed between poor dietary knowledge and unhealthy eating behaviors. Similarly, dietary attitudes were strongly associated with healthy dietary behaviors when the effects of other factors were excluded; responders with negative attitudes were more likely to exhibit unhealthy eating behaviors.

**Conclusion:**

Our findings suggest that residents in the Northeast China possessed a relatively low level of nutritional knowledge, which directly influenced their dietary practices during the COVID-19 pandemic. This study provides valuable insights into the cross-sectional description and key factors related to nutrition-related KAP, serving as a basis for future policymaking to respond more effectively to health crises.

## Background

The COVID-19 pandemic, caused by the severe acute respiratory syndrome coronavirus 2 (SARS-CoV-2), emerged in late 2019 and has since evolved into the Omicron strain (1–3). SARS-CoV-2 enters target cells by binding to the angiotensin-converting enzyme 2 (ACE2) through the spike protein receptor-binding domain (RBD) (4, 5). COVID-19 infections can manifest as asymptomatic or mild cases, characterized by symptoms like cough, chills, fever, fatigue, and shortness of breath, typically without pneumonia (6). However, severe cases, marked by pneumonia, often involve an immune system imbalance, leading to an influx of inflammatory cells, including monocytes and neutrophils (7). This process causes the loss of alveolar epithelial cells, impairing barrier function and leading to alveolar edema, oxygen exchange dysfunction, and potentially pneumonia or acute respiratory distress syndrome (ARDS). Similar pathological damage occurs in other target organs, such as the liver and myocardium, with the release of cytokine storms and even fatalities (6, 8).

As of April 2022, COVID-19 had claimed ~6.11 million lives globally, with an estimated excess death toll reaching 18.2 million. The pandemic's impact extends beyond the reported death toll, affecting healthcare systems, mental health, lifestyles, and socioeconomic factors (9, 10). Despite the deployment of effective SARS-CoV-2 vaccines in many countries, uneven vaccine coverage has not always halted virus transmission, leaving the global COVID-19 epidemic a persistent threat (11). Predicting the evolution of the virus toward greater or lesser virulence remains challenging, although Omicron has caused milder disease, it still poses significant immune escape challenges (11–15). Consequently, a future with a high prevalence of SARS-CoV-2 infections is likely to persist.

Nutrition is a vital determinant of overall health and plays a crucial role in the prognosis of individuals infected with SARS-CoV-2 (16, 17). Adequate energy and protein intake are essential for improving nutritional status and immune function, potentially aiding in infectious disease recovery (18). Notably, systemic inflammation may exacerbate COVID-19, explaining the increased mortality and severity in individuals with pre-existing health conditions like chronic lung diseases, obesity, liver disease, diabetes, and chronic kidney disease (19, 20). Unhealthy dietary habits can contribute to chronic diseases by promoting low-grade inflammation and oxidative stress (21). Regional variations in dietary habits, influenced by climate and culture, further contribute to health disparities (22, 23). Research has consistently shown the ability of nutrition to influence immune system function (24–26). Given the lack of specific drugs for SARS-CoV-2 and incomplete vaccination coverage, optimizing nutrition appears to be one of the most cost-effective means of enhancing immunity (18). However, assessing nutritional status is just one aspect; understanding what and how people eat and the factors underlying their dietary habits is essential. The knowledge-attitude-practice (KAP) model is the most commonly used model to explain how individual knowledge and attitudes affect health behavior. Previous studies have indicated that nutritional knowledge positively impacts the adoption of healthy eating habits (27). Also, people with positive attitudes are more likely to make healthier dietary practices than people with negative attitudes (28, 29). Therefore, increasing the nutrition-related knowledge and improving nutritional attitudes is very important during the COVID-19 Era. Although evaluating people's nutrition-related knowledge, attitudes, and practices offers insights into the personal determinants of dietary behavior (18), no study was conducted to explore the nutrition-related KAP and related influencing factors for dietary behaviors among residents in China during the COVID-19 epidemic.

Thus, this study aimed to fill the gap in our understanding by conducting a systematic investigation into the KAP of residents in Northeast areas of China. In addition, we also explored the factors influencing dietary behaviors through logistic regression analysis. Our findings can inform health experts and policymakers in designing nutrition care programs that promote public health during the ongoing COVID-19 pandemic.

## Materials and methods

### Study design and participants

This project was a nationwide survey at subsidized by Chinese Nutrition Society. There are eight geographically distinct regions in China, including Northeast region, East region, North region, South region, Central region, Northwest region, southwest region, and Hongkong-Macao. For this study, we focused on the Northeast region, which includes Liaoning Province, Jilin Province, and Heilongjiang Province, covering an area of 0.78 million square kilometers with a population of ~110 million. The Northeast region's unique geographical location (borders Russia and Korea), historical culture (occupied by Russia and Japan for a long time), and environmental characteristics (famous for its agriculture and fish) set it apart from other parts of China. Notably, the residents in this region exhibit distinctive dietary and lifestyle behaviors due to these factors.

We implemented this survey in four major cities within the Northeast region: Shenyang (the capital of Liaoning), Changchun (the capital of Jilin), Harbin (the capital of Heilongjiang), and Dalian (a significant economic center and port city). This cross-sectional study was conducted between January and December 2022 using a self-administered questionnaire. We employed a snowball sampling strategy, initially recruiting 50 participants with diverse professional backgrounds in each city. Subsequently, these participants were encouraged to refer the same questionnaire to their relatives and friends for data collection. The survey maintained anonymity, and participation was voluntary. Eligible respondents were healthy individuals aged at least 18 years, residing locally. We excluded individuals who (1) were aged <18 years; (2) couldn't complete the questionnaire appropriately; (3) afflicted by illnesses that could affect their dietary behaviors.

### Data collection

The questionnaire, developed by experts from the Chinese Nutrition Society based on the Dietary Guidelines for Chinese Residents (2020), underwent modifications to align with the study's objectives, particularly with input from experts in the Dalian cities. The questionnaire, comprising of fours parts. The first parts were about respondents' sociodemographic information (*n* = 10), including age, sex, education level, income, location, ethnicity, occupation, respondents' height and weight. The second part consisted of 12 questions and investigated the knowledge about Dietary Guidelines for Chinese Residents (2020). The third part used 10 questions to gather the data about reasonable dietary attitudes. The final part of the questionnaire asked about dietary behaviors. It consisted of 7 questions aimed at investigating diet-related practices during COVID-19 epidemic. The construct validity and reliability analyses were performed on the 200 people using 29 questionnaire items including nutrition-related knowledge, attitudes, and practices. Reliability and validity were calculated through Cronbach's alpha statistical test and Kaiser–Meyer–Olkin test, respectively. The high Cronbach's Alpha of 0.94 (*P* < 0.05) and the Kaiser–Meyer–Olkin test of 0.92 (*P* < 0.05), confirmed the internal reliability and excellent validity of the questionnaire.

### Statistics

Data were double-entered and cleaned using Epidata 3.02, with SPSS 26.0 (SPSS Inc., Chicago, IL) used for logical error detection and initial data validation. All the data were analyzed qualitatively and were expressed as a percentage (%) and numbers (*n*). Knowledge questions were multiple-choice with a single correct answer. Each correct answer was given one point, while an incorrect answer was given zero point. Nutritional knowledge scores were computed by summing responses to each question, classifying participants with scores ≥60% as having good knowledge and those with scores <60% as having poor knowledge. The score of nutrition-related attitudes were assessed on a Likert Scale scored from 0 to 4. Study participants with total scores of ≥21 points were categorized as having a positive attitude, while others were considered to have a negative attitude. Each participant was also asked to indicate how often he or she exhibited each of seven nutrition-related behaviors on a 5-point scale that ranged from “never” to “always”; and study participants were classified to have heathy dietary practices if they get a total score of reasonable dietary practices ≥17 points. Categorical variables were analyzed using the chi-squared test. Logistic regression analysis was applied to estimate the adjusted odds ratio (AOR) and their 95% confidence intervals (95% CI) for dependent and independent variables. Binary logistic regression analysis was applied to estimate the corresponding influential factors of dietary behavior healthy or not (dependent variable categorized as 0 = unhealthy dietary behavior; 1 = healthy dietary behavior). All test were 2-side and a *P* < 0.05 was considered statistically significant.

Family income was classified into six categories (<5000 yuan, 5000–9,999 yuan, 10,000 yuan −19,999 yuan, 20,000–39,999 yuan, 40,000–79,999 and ≥80,000 yuan), age into six categories (<25, 25–29, 30–39, 40–49, 50–59 and ≥60), and education into six categories (junior school or below, junior middle school, high school degree, postsecondary degree, college degree, and postgraduate or above). Body mass index (BMI) was computed as the ratio of weight in kilograms to heights in meters squared and categorized as obese (BMI ≥ 30), overweight (BMI between 25–29.9), normal weight (BMI between 18.5–24.9), and underweight (BMI <18.5).

## Results

### Characteristics of the participants and weight status based on BMI category

A total of 4,092 respondents completed the questionnaires in our study. Twenty-seven respondents were excluded from the analysis due to missing or erroneous information. The final analysis included 4,065 participants, with 1,005 from Dalian, 1,009 from Shenyang, 1,049 from Changchun, and 1,002 from Harbin. The mean age of the respondents was 39.76 ± 12.35 years, 23.6% respondents were younger than 30 years, 55.2% respondents were between 30 and 49 years, and 21.2% of respondents were older than 50 years. Due to the nature of certain workplaces, such as agricultural, animal husbandry, or fishing, there was a higher representation of men than women in our study. Regarding education, among the 4,065 participants interviewed, 14.5% had a high school below, 30.6% had a high school degree, 29.3% had a postsecondary degree, 20.2% had a college degree and 5.5% had a postgraduate education or above. A significant proportion of respondents had a monthly family income of 10,000 yuan−39,999 yuan. The majority of participants had a normal BMI; 33.5% participants had BMI between 25 and 29.9 and 5.2% had BMI more than 30. Descriptive characteristics of the study participants are shown in Table 1.

**Table 1:** Demographic characteristics of the participants (*N* = 4,065).

**Characteristic**		***N***	**%**
City of residence	Dalian	1,005	24.7
	Harerbin	1,002	24.6
	Shenyang	1,009	24.8
	Changchun	1,049	25.8
Gender	Male	2,613	64.3
	Female	1,452	35.7
Age (years)	<25	336	8.3
	25–29	622	15.3
	30–39	1,427	35.1
	40–49	819	20.1
	≥50	861	21.2
Ethnicity	Han	3,783	93.1
	Meng	139	3.4
	Others	143	3.5
Occupation	Farmer	978	24.1
	Students	477	11.7
	Not Employed	245	6.0
	Freelance	679	16.7
	Employed Public institution	1,153	28.4
	Salesman	533	13.1
Education level	Junior middle school or below	589	14.5
	High school	1,243	30.6
	Postsecondary degree	1,190	29.3
	College degree or above	1,043	25.7
Family monthly income (¥)	<5000	627	15.4
	5000–9999	827	20.3
	10,000–19,999	1,085	26.7
	≥20,000	1,526	37.5
BMI	Underweight	269	6.6
	Normal weight	2,434	59.9
	Overweight	1,152	28.3
	Obese	209	5.2

BMI, body mass index.

### Dietary knowledge of study participants

The dietary knowledge questions were developed based on the Dietary Guidelines for Chinese Residents (2020). Correct answer rates ranged from 18.3% to 73.8%. Notably, 68.1% of respondents correctly identified the relationship between salt intake and hypertension, while 73.8% of the study participants correctly recognized the importance of “Eat a variety of foods, with cereals as the staple.” However, knowledge on other variables was less satisfactory, with fewer than 60% of respondents answering correctly. Only one-third of the respondents were knowledgeable about the daily levels of oil, salt, milk, water, vegetables and fruits for adults (items K1-K5). Overall, our findings indicated that participants had inadequate nutrition knowledge (Table 2).

**Table 2:** Percentage of correct answers related to dietary knowledge (*N*/%).

**Items**	**Correct answer**		**Wrong answer**	
	***N***	**%**	***N***	**%**
K1	1,346	33.1	2,719	66.9
K2	1,120	27.6	2,945	72.4
K3	1,478	36.4	2,587	63.6
K4	1,249	30.7	2,816	69.3
K5	1,508	37.1	2,557	62.9
K6	820	20.2	3,145	79.8
K7	1,905	46.9	2,160	53.1
K8	743	18.3	3,322	81.7
K9	1,831	45	2,234	55
K10	2,767	68.1	1,298	31.9
K11	3,000	73.8	1,065	26.2
K12	2,162	53.2	1,903	46.8

Dietary knowledge questionnaire: K1: How many grams of cooking oil are recommended for adults per day according to Chinese Dietary Guidelines (2020)? K2: How many grams of salt are recommended for adults per day according to Chinese Dietary Guidelines (2020)? K3: How many grams of milk are recommended for adults per day according to Chinese Dietary Guidelines (2020)? K4: How many liters of water are recommended for adults per day according to Chinese Dietary Guidelines (2020)? K5: How many grams of vegetables and fruits are recommended for adults per day according to Chinese Dietary Guidelines (2020)? K6: How many kinds of food are recommended for adults per day according to Chinese Dietary Guidelines (2020)? K7: Which kind of food do you think is the best sources of calcium? K8: What kind of food would you like to eat except for egg, milk, and a portion of vegetables for breakfast? K9: Does a healthy diet require serving of individual dishes? K10: Is the salt intake closely related to the incidence of hypertension? K11: What kind of food serves as the staple based on the food consumption recommended in the Food Pagoda? K12: Which vitamin deficiency is related to nyctalopia or night blindness?

Each correct answer was given one point, while an incorrect answer was given zero. The score of nutritional knowledge was calculated by summation of responses of each question. Out of 12 knowledge questions, the median knowledge score was 5, with Q25–Q75 ranging from 3 to 7. Subsequently, we analyzed the pass rate (correctly answer ≥ 60% of questions) of the respondents' dietary knowledge. Our results showed that pass rate was only 26.5% in this survey. Only 0.8% of the respondents scored ≥ 80% of the correct answers. The pass rate varied among different demographic groups (Supplementary Table 1).

### Attitudes toward healthy eating of study participants

In the attitudes toward healthy eating part, there were ten questions shown in Table 3. Notably, 47.6% of respondents mistakenly believed that wildlife is more nutritious; 25.5% of respondents agreed that fast food has no adverse health effects; additionally, up to 42% of participants believed that drinking sugar-sweetened beverages could replace water, and 40.8% thought that consuming brown sugar could treat anemia. For further analysis, items (Q1-Q2, Q4-Q10) were rated on a 5-point Likert scale from “strongly agree” to “strongly disagree,” with numeric scores ranging from zero to four. For reverse items (Q3), the scores were re-coded as four, three, two, one, and zero, with “strongly agree” scoring of four. Summary scores were used to describe levels of healthy eating attitudes. Participants were classified as having a positive attitude if they scored ≥21 points; otherwise, they were considered to have a negative attitude. Our results showed that only 60.6% of participants in this survey held positive attitudes toward healthy eating.

**Table 3:** Dietary attitudes among the residents during the COVID-19 pandemic (*N*/%).

**Items**	**Strongly agree**	**Agree**	**Neutral**	**Disagree**	**Strongly disagree**
Q1	94 (2.3%)	569 (14.0%)	1,245 (30.6%)	991 (24.4)	1,166 (28.7%)
Q2	496 (12.2%)	1,488 (36.6%)	985 (24.2%)	838 (20.6%)	258 (6.3%)
Q3	44 (1.1%)	366 (9.0%)	770 (18.9%)	2,029 (49.9%)	856 (21.1%)
Q4	559 (13.8%)	1,509 (37.1%)	1,139 (28.0%)	707 (17.4%)	151 (3.7%)
Q5	1,123 (27.6%)	1,473 (36.2%)	1,183 (29.1%)	168 (4.1%)	118 (2.9%)
Q6	36 (0.9%)	241 (5.9%)	691 (17.0%)	1,803 (44.4%)	1,294 (31.8%)
Q7	41 (1.0%)	218 (5.4%)	689 (16.9%)	1,769 (43.5%)	1,348 (33.2%)
Q8	842 (20.7%)	866 (21.3%)	1,070 (26.3%)	564 (13.9%)	723 (17.8%)
Q9	414 (10.2%)	1,245 (30.6%)	1,295 (31.9%)	663 (16.3%)	448 (11.0%)
Q10	44 (1.1%)	370 (9.1%)	613 (15.1%)	1,556 (38.3%)	1,482 (36.5%)

Dietary attitudes questionnaire: Q1: Wildlife is more nutritious. Q2: Vegetarian diet is more beneficial to health than balance portion of vegetables and meat. Q3: It is appropriate to eat moderate. Q4: Fast food has no adverse health effects. Q5: Traditional Chinese medicine has been documented as a safe treatment for preventive and/or chronic disease and should be completely reserved. Q6: You can enjoy delicious food during the holidays without considering your health problems. Q7: You can have a midnight snack daily if you keep exercising. Q8: You can drink a lot sugar-sweetened beverage instead of water. Q9: Brown sugar is good for the treatment of anemia. Q10: Don't need use serving chopsticks and spoons when you eat with your family.

### Dietary behaviors of study participants during the pandemic

Table 4 summarizes the dietary behaviors of study participants during the COVID-19 pandemic. During quarantine, only 10.8% of participants read nutrition labels when purchasing prepackaged foods either most of the time or always; only one-third of the respondents (33.1%) prepared raw and cooked food separately either most of the time or always; the majority of the participants didn't eat out at restaurants/order takeaways during the COVID-19 pandemic; under half of the participants (44.2%) packed the leftovers if they ate out at restaurants either most of the time or always; 14.9% of the surveyed participants plied others to drink a lot and 13.6% washed hands before meal either most of the time or always; only a minority of the participants ate wild animals either most of the time or always during the COVID-19 pandemic; the majority of the participants consumed home-cooked food; 14.4% of the participants bought vegetables and fruits from fresh supermarket; only 17.5% of the participants like bland food. For further analysis, the response choices and their designated scores and points were as follows: “Never” = 0; “Occasionally” = 1; “Sometimes” = 2; “Most of times” = 3; “Never” = 4. These choices and points were applied for the first four questions (Q1–Q4). For other items (Q5–Q7), the scores were re-coded as four, three, two, one, and zero with the answer “never” receiving a score of four. For item Q8:Where do you usually consume food?” the response choices and their designated scores were as follows: “eating home-cooked”= 2; “eating at the canteen”= 1; “eating at restaurants”= 0; For item Q9, the participants bought vegetables and fruits “from supermarket,” “from fresh supermarket,” “from famer's market,” and “from street market” received three, two, one, and zero points, respectively; For item Q10, the participants reported liking bland food were given one point, while other answers were given zero point. The total score of this section corresponded to the sum of the scores in the ten questions. The total score of diet behaviors is from “0” to “34,” where “0” designates severe unhealthy dietary behaviors and “34” designates no unhealthy dietary behaviors. Out of 10 dietary behaviors questions, the median score was 17, with Q25-Q75 ranging from 15 to 21. Furthermore, among the 4,092 participants, only 54.6% of the participants have heathy dietary practices (got a total score of reasonable dietary practices ≥17 points) during COVID-19 pandemic.

**Table 4:** Dietary behaviors of study participants during the COVID-19 pandemic (*N*/%).

**Items**	**Never**	**Occasionally**	**Sometimes**	**Most of times**	**Always**
Q1	728 (17.9)	1788 (44.0)	935 (23.0)	474 (11.7)	140 (3.4)
Q2	385 (9.5)	1,143 (28.1)	1,191 (29.3)	991 (24.4)	355 (8.7)
Q3	1,579 (38.8)	1,783 (43.9)	358 (8.8)	266 (6.5)	79 (1.9)
Q4	710 (17.5)	1,947 (47.9)	805 (19.8)	438 (10.8)	165 (4.1)
Q5	830 (20.4)	1,195 (29.4)	1,490 (36.7)	486 (12.0)	64 (1.6)
Q6	212 (5.2)	926 (22.8)	1,128 (27.7)	1,086 (26.7)	713 (17.5)
Q7	1,364 (33.6)	1,202 (29.6)	1,046 (25.7)	290 (7.1)	163 (4.0)

Dietary behaviors questionnaire: Q1: How often do you read nutrition labels when you purchase prepackaged foods? Q2: How often do you prepare raw and cooked food separately? Q3: How often do your family eat out at restaurants/order takeaways? Q4: How often do you pack the leftovers when you eat out at restaurants? Q5: How often do you urge others to drink a lot? Q6: How often do you wash your hands before meal? Q7: How often do you eat wild animals (gamey)? Q8: Where do you usually consume food? Q9: Where did you buy vegetables and fruits? Q10: What flavor food do you like best?

### Influence of dietary knowledge and attitudes on dietary behaviors

The chi-squared test was employed to assess differences in dietary behaviors according to demographic characteristics (Table 5). Our results showed that nutritional knowledge was positively associated with dietary behaviors. Participants with good knowledge exhibited a higher adherence to healthy dietary behaviors compared to those with poor knowledge (74.6% vs. 50%). Moreover, participants' dietary attitudes significantly influenced their dietary behaviors. Participants with a positive attitude toward healthy diet were more likely to adhere to healthy eating behaviors than those with a negative dietary attitude (68.2% vs. 33.8%). In addition, socioeconomic characteristics including geographical area, age, gender, ethnicity, family monthly income, education levels, and occupation, were significantly associated with participants' dietary behaviors. However, there were no significant differences in dietary behaviors of participants based on BMI and residence.

**Table 5:** Univariate analysis of dietary behaviors of study participants.

**Characteristics**		**Healthy behaviors (*N*/%)**	**χ^2^**	***P*-value**
Residence	Dalian	657 (65.4)	97.18	0.00
	Haerbin	523 (52.2)		
	Shenyang	444 (44.0)		
	Changchun	596 (56.8)		
Age (years)	<25	196 (58.3)		
	25–29	357 (57.4)	14.11	0.007
	30–39	783 (54.9)		
	40–49	459 (56.0)		
	≥50	425 (49.4)		
Gender	Male	1,305 (49.9)	64.36	0.00
	Female	537 (63.0)		
Ethnicity	Han	2,075 (54.9)	22.948	0.00
	Meng	52 (37.4)		
	Others	93 (65.0)		
Education level	Junior middle school or below	365 (62.0)		
	High school	531 (42.7)	287.76	0.00
	Postsecondary degree	548 (46.1)		
	College degree or above	776 (74.4)		
Family monthly income (¥)	<5000	407 (64.9)		
	5000–9999	510 (61.7)	91.68	0.00
	10,000–19,999	604 (55.7)		
	≥20,000	699 (45.8)		
Occuption	Farmer	597 (61.0)		
	Students	171 (35.8)		
	Not Employed	1,80 (73.5)	189.18	0.00
	Freelance	296 (43.6)		
	Employed Public institution	719 (62.4)		
	Salesman	257 (48.2)		
BMI	Underweight	145 (53.9)	3.27	0.353
	Normal	1,304 (53.6)		
	Overweight	653 (56.7)		
	Obesity	117 (56.0)		
Knowelege score	Poor	1,650 (50.0)	151.75	0.00
	Good	570 (74.6)		
Dietary attitude	Negative	542 (33.8)	463.42	0.00
	Positive	1,678 (68.2)		

To further evaluate the association of dietary knowledge and attitudes with socio-demographic determinants and their potential impact on healthy eating behaviors, we employed a binary logistic regression model, with the dependent variable categorized as 1 for unhealthy dietary behaviors and 0 for healthy dietary behaviors. We included several potential confounding variables in the analysis, such as socioeconomic characteristics, BMI, dietary knowledge scores categorized as poor or good knowledge, and dietary attitudes categorized as positive or negative attitudes. The results presented in Table 6 highlight the factors influencing dietary behaviors among the 4,065 respondents. Compared to respondents residing in Dalian city, those living in Harbin (AOR = 1.25; 95% CI: 1.01–1.55), Shenyang (AOR = 1.81; 95% CI: 1.46–2.24), and Changchun (AOR = 1.08; 95% CI: 0.83–0.32) exhibited a higher likelihood of unhealthy eating behaviors. Men were more inclined to display adverse dietary behaviors compared to women (AOR = 1.57; 95% CI: 1.34–1.84). Respondents with lower education levels were also more prone to unhealthy dietary behaviors. The proportion of individuals with unhealthy dietary behaviors was higher among Mongolian ethnic respondents compared to Han ethnic individuals (AOR = 1.97; 95% CI: 1.32–2.93), with Mongolian ethnic respondents being 1.97 times more likely to exhibit unhealthy dietary behaviors. Family income was significant negative association with healthy dietary behaviors; respondents with family incomes of 10,000–19,999 (AOR = 1.51; 95% CI: 1.19–1.92) and more than 20,000 (AOR = 1.74; 95% CI: 1.38–2.18) were more likely to exhibit unhealthy eating behaviors compared to those with lower family incomes. Moreover, working respondents and students were more prone to unhealthy eating behaviors compared to farmers. Age and BMI weakly influence dietary behaviors when the effects of other factors were excluded. Importantly, the strongest associations were observed between poor dietary knowledge (AOR = 2.41; 95% CI: 2.02–2.87) and unhealthy eating behaviors. Similarly, the results also indicated that dietary attitudes were strongly positively associated with healthy dietary behaviors when the effects of other factors were excluded; responders with negative attitudes were more likely to exhibit unhealthy eating behaviors (AOR = 3.47; 95%CI: 2.99–4.03).

**Table 6:** Adjusted odds ratio with 95% confidence intervals for dietary behaviors and corresponding influential factors (logistic regressions).

**Variable**	**B**	**S.E**.	**Waldχ^2^**	**df**	***P*-value**	**AOR**	**95%CI**	
							**Lower**	**Upper**
**City** (Con = Dalian)			37.02	3	0.00			
Haerbin	0.23	0.11	4.266	1	0.04	1.25	1.01	1.55
Shenyang	0.593	0.108	29.959	1	0.000	1.809	1.463	2.237
Changchun	0.072	0.106	0.465	1	0.495	1.075	0.873	1.323
**Gender** (Con = female)	0.450	0.081	30.894	1	0.000	1.568	1.338	1.837
Ethnicity (Con =Dalian)			11.702	2	0.003			
Meng	0.677	0.204	11.028	1	0.001	1.968	1.320	2.934
Others	−0.130	0.202	0.417	1	0.519	0.878	0.591	1.304
**Age (years)** (Con <25)			5.870	4	0.209			
25–29	0.358	0.164	4.735	1	0.030	1.430	1.036	1.974
30–39	0.291	0.152	3.655	1	0.056	1.337	0.993	1.801
40–49	0.271	0.163	2.760	1	0.097	1.311	0.953	1.804
≥50	0.361	0.162	4.941	1	0.026	1.435	1.044	1.973
**Education** (Con = College degree or above)			161.634	3	0.000			
Junior middle school or below	0.847	0.138	37.624	1	0.000	2.332	1.779	3.056
High school	1.330	0.112	140.468	1	0.000	3.782	3.035	4.712
Postsecondary degree	1.106	0.105	110.992	1	0.000	3.024	2.461	3.715
**Occupation** (Con = Farmer)			99.793	5	0.000			
Students	1.252	0.145	74.819	1	0.000	3.496	2.633	4.642
Not Employed	−0.272	0.176	2.385	1	0.122	0.762	0.540	1.076
Freelance	0.604	0.117	26.614	1	0.000	1.829	1.454	2.301
Employed Public institution	0.396	0.112	12.572	1	0.000	1.485	1.194	1.849
Salesman	0.417	0.127	10.825	1	0.001	1.517	1.184	1.945
**Monthly income** (yuan) (Con ≤ 5000)			38.842	3	0.000			
5000–9999	0.022	0.130	0.028	1	0.867	1.022	0.792	1.319
10,000–19,999	0.410	0.122	11.226	1	0.001	1.507	1.186	1.916
≥20,000	0.551	0.116	22.445	1	0.000	1.735	1.381	2.180
**BMI** (Con = Obesity)			8.193	3	0.042			
Underweight	0.020	0.219	0.008	1	0.927	1.020	0.664	1.567
Normal	−0.011	0.168	0.005	1	0.946	0.989	0.711	1.375
Overweight	−0.245	0.175	1.960	1	0.161	0.783	0.556	1.103
**Knowledge score** (Con = Good)	0.878	0.090	95.789	1	0.000	2.407	2.018	2.869
**Dietary attitude** (Con = Positive)	1.245	0.076	268.931	1	0.000	3.473	2.993	4.031
Constant	−6.265	0.345	329.624	1	0.000	0.002		

## Discussion

People pay more attention to reduce the spread of the virus at the beginning of COVID-19 pandemic. Soon, several studies indicated that it is very important to attain good nutritional status of individuals to build a robust immune system to fight against COVID-19 (30, 31). To our knowledge, this study was first to investigate the nutrition-related KAP among residents in the Northeast regions of China during the COVID-19 epidemic. KAP prevalence survey has the potential to uncover the underlying causes that hinder healthy dietary behaviors and provide valuable insights to guide interventions aimed at rectifying such behaviors.

According to the KAP model, nutritional knowledge can significantly influence dietary practices through attitudes, and these practices, in turn, shape dietary patterns, ultimately impacting an individual's nutrient intake (32, 33). Our study revealed a very good knowledge of nutrition and the role it plays in maintaining healthy. Therefore, fostering correct nutrition-related KAP is crucial, especially in a period when the immune system needs to fight back. Our study showed that nutrition-related KAP among residents in Northeast China was suboptimal, indicating a need for targeted educational measures.

Firstly, our findings indicate that respondents had limited understanding of rational diets and dietary guidelines. Among the 12 questions related to nutritional knowledge, the first five items, which focused on recommended daily intake (RDI) of common foods (such as cooking oil, milk, salt, water, vegetables, and fruits), had an average percentage of the correct answers in our study only around 33% for each. These answers are explicitly stated in the Dietary Guidelines for Chinese Residents (2020) and the “Dietary Guidelines for the Prevention and Treatment of Novel Coronavirus” published by the National Health Commission of China (34, 35). Notably, the respondents showed better grasp of nutritional knowledge related to food functions, such as the relationships between salt and high blood pressure, vitamin A and night blindness, and cereals and dietary patterns. However, the study data suggest that there is room for improvement in providing clear and comprehensive food quantitative guidance to the public. Attention should be paid to the traditional eating habits tend to be high in fat, sugar, and salt with a high dietary energy, fat and meat among residents in the Northeast regions of China. Therefore, provision of correct diet education is essential to help people to maintain nutritional status and immune function, potentially aiding in infectious disease recovery.

Secondly, our results showed that only 60.6% of participants in this survey held positive attitudes toward healthy eating, the findings on some of these issues were noteworthy. For example, nearly half of the respondents still believed that “wild animals are more nutritious.” As a response to the COVID-19 epidemic, China has enforced a legal ban on the consumption of terrestrial wildlife (36). While it's true that wild animals may have high nutritional value due to their amino acid and fatty acid profiles (37–39), they are also susceptible to consuming poisonous and pathogen-infected food, posing health risks to consumers (40–45). Therefore, stricter laws prohibiting the slaughter and trade of wild animals should be enacted and enforced. Additionally, the risks associated with consuming wild animals should be widely publicized. Interestingly, almost half of the respondents in our study agreed that “a vegetarian diet is healthier than a balanced diet of meat and vegetables.” Although vegetarian diets have demonstrated benefits in various health aspects, they may also lead to deficiencies in essential nutrients such as protein, omega-3 fatty acids, vitamin D, vitamin B12, iron, calcium, and zinc (46). Therefore, while vegetarian diets are gaining popularity, it is essential to emphasize balanced diets in dietary guidelines (47). Similarly, more than 50% of respondents agreed that “eating takeaway food does not affect your health.” Over the past few years in China, with the rapid development of Internet, mobile phone and online payment, takeaway food has increased rapidly. Takeaway meals often contain higher levels of energy, fat, sugar, and salt but fewer vitamins and minerals than home-cooked meals (48–50), contributing to overweight or obesity and chronic diseases (51–53).Interestingly, a majority of the studies reported that people increased consumption of fast foods, sweet snacks, and sugar-sweetened beverages in the COVID-19 era (12, 54). However, takeaway food delivery services play a crucial role in providing job opportunities and supporting businesses, especially during epidemics. The challenge is to transform takeaway food options to be more balanced and healthier.

In terms of dietary practices, we found that more than 60% of respondents reported that they seldom or never read nutrition labels on packaged foods and drinks. This behavior reflects a lack of nutrition knowledge, as nutrition labels provide important information for making healthier food choices (55). Given that understanding these labels requires both basic nutritional knowledge and numeracy skills, implementing Front-of-Pack (FOP) labeling strategies that provide key nutrition information in an easily understandable format is recommended (56–58). Furthermore, our survey highlighted that some critical practices related to food hygiene and safety were not consistently followed by respondents. For instance, nearly 40% of respondents did not separate raw food from other foods during food preparation, and nearly 30% did not wash their hands before meals. About one-third of the respondents mistakenly believed that it's not necessary to use serving chopsticks and spoons when eat with family. Similar to our data, a study in Chinese residents showed that less than half participants used serving chopsticks and spoons and prepared raw and cooked food separately in the COVID-19 Era (59). In order to reduce the risk of contracting the COVID-19, Chinese professionals recently encourage people to use serving chopsticks and spoons and to adopt individual food services (59). However, these dietary practices are very different from traditional Chinese customs. Thus, these recommendations regarding food safety and hygiene should be incorporated in the current health education, especially during the COVID-19 pandemic.

Binary logistic regression models identified significant differences in dietary behaviors based on factors such as gender, ethnicity, educational level, occupation, household monthly income, and BMI. Our results showed that the following characteristics were associated with healthy eating behaviors: being resided in Dalian city, being female, having a higher education level, having a higher family income, and being employed by government employees and teachers. These factors are reflective of socioeconomic status and health literacy. Higher education levels have been associated with better nutrition-related KAP, which was also supported by our findings. Our findings are consistent with that of previous studies, in which educational status and occupation were factors to affect dietary practice (28, 60, 61). In most cases, nutrition knowledge can attenuate socioeconomic differences in food purchase choices, and nutrition education efforts are best focused on raising awareness of the relationship between diet and disease among low level of income and education (62). Our results were also in line with an Italian survey, optimizing nutrition-related knowledge, dietary habits and lifestyle is still urgently needed and should be continued during the ongoing COVID-19 pandemic (54). Unsurprisingly, our study indicated that both good dietary knowledge and a positive dietary attitude can strongly promote healthy dietary practice.

### Limitations

Several potential limitations should be addressed for this study. First, our study used a snowball sampling strategy due to the COVID-19 pandemic. The snowball strategy relies on participants referring others, such as relatives and friends, to participate in the study. This sampling method may introduce biases into the sample and compromise the representativeness of the population under study. Second, respondents in this study were associated with well-established sea cucumber enterprises, possibly affecting their economic status and introducing potential bias. Third, the study did not assess the nutritional status of respondents directly, limiting the ability to draw direct health implications from nutrition-related KAP.

## Conclusion

In this study, we have provided the data related to nutrition-related KAP among residents in the Northeast China during the COVID-19 pandemic for the first-time, thus contributing to the identification of potential targets for nutritional interventions. Our findings confirmed that high nutrition-related knowledge and positive attitudes were strongly associated with positive nutrition-related health seeking practices. It was worth noting that a relatively low level of nutritional knowledge, negative attitudes and unhealthy dietary behaviors still existed in a large proportion of participants. Thus, our findings may also inform public health officials on proper nutritional education and knowledge about adoption of a well-balanced nutritional model, food safety, healthy practices, and policy improvements pertaining to the COVID-19 outbreak.

## Data availability statement

The original contributions presented in the study are included in the article/Supplementary material, further inquiries can be directed to the corresponding authors.

## Ethics statement

Ethical review and approval was not required for the study on human participants in accordance with the local legislation and institutional requirements. Written informed consent from the patients/participants was not required to participate in this study in accordance with the national legislation and the institutional requirements.

## Author contributions

LH: Conceptualization, Formal analysis, Funding acquisition, Writing—original draft. XX: Conceptualization, Investigation, Methodology, Writing—review & editing. YD: Conceptualization, Investigation, Methodology, Writing—review & editing. YZ: Investigation, Writing—review & editing. SL: Investigation, Writing—review & editing. WL: Investigation, Writing—review & editing. JZ: Conceptualization, Resources, Writing—review & editing. KW: Conceptualization, Writing—review & editing. LZ: Conceptualization, Writing—review & editing. QW: Conceptualization, Project administration, Supervision , Resources, Writing—review & editing.
