# Supplementary material for: Nutritional knowledge, attitudes, and practices among residents in the Northeast areas of China during the COVID-19 epidemic
Source: Front Public Health. 2024 Jan 30;12:1296869. doi: 10.3389/fpubh.2024.1296869 (PMC10861797; doi:10.3389/fpubh.2024.1296869)
Supplement: Supplementary file 1 [file Table_1.DOCX]

**Supplementary Table 1 |** Comparison of knowledge scores amongst different demographic groups（N=4065）

| **Characteristics** |  | **Pass**  **N** | **Total** | **Passed (%)** | ***P*-Value** |
| --- | --- | --- | --- | --- | --- |
| **City of residence** | Dalian | 267 | 738 | 267(26.6) | 0.026 |
|  | Haerbin | 297 | 705 | 297(29.6) |  |
|  | Shenyang | 239 | 770 | 239(23.7) |  |
|  | Changchun | 273 | 776 | 273(26.0) |  |
| **Age (years)** | ﹤25 | 78 | 258 | 78(23.2) | 0.00 |
|  | 25-29 | 151 | 471 | 151(24.3) |  |
|  | 30-39 | 343 | 1084 | 343(24.0) |  |
|  | 40-49 | 275 | 544 | 275(33.6) |  |
|  | ≥50-59 | 229 | 632 | 229(36.2) |  |
| **Gender** | Male | 651 | 1962 | 651(24.9) | 0.003 |
|  | Female | 425 | 1027 | 425(29.3) |  |
| **Ethnicity** | Han | 1024 | 2759 | 1024(27.1) | 0.00 |
|  | Meng | 15 | 124 | 15(10.8) |  |
|  | Others | 37 | 106 | 106(25.9) |  |
| **Education level** | Junior middle school or below | 141 | 589 | 141(23.9) | 0.00 |
|  | High school | 269 | 974 | 269(21.6) |  |
|  | Postsecondary degree | 327 | 863 | 327(27.5) |  |
|  | College degree or above | 339 | 704 | 339(32.5) |  |
| **Family monthly Income (¥)** | <5000 | 99 | 627 | 99(15.8) | 0.00 |
|  | 5000-9999 | 327 | 827 | 327(39.5) |  |
|  | 10,000-19,999 | 364 | 1085 | 364(33.5) |  |
|  | ≥20,000 | 286 | 1526 | 286(18.7) |  |
| **Occupation** | Farmer | 195 | 978 | 195(19.9) | 0.00 |
|  | Students | 71 | 477 | 71(14.9) |  |
|  | Not Employed | 71 | 245 | 71(29.0) |  |
|  | Freelance | 168 | 679 | 168(24.7) |  |
|  | Employed Public institution | 429 | 1153 | 429(37.2) |  |
|  | Salesman | 142 | 533 | 142(26.6) |  |
| **BMI** | Underweight | 69 | 200 | 69(25.7) | 0.822 |
|  | Normal | 642 | 1792 | 642(26.4) |  |
|  | Overweight | 303 | 849 | 303(26.3) |  |
|  | Obesity | 61 | 148 | 61(29.2) |  |
